# Assessment of Selected Aspects of the Quality of Life of Children with Type 1 Diabetes Mellitus in Poland

**DOI:** 10.3390/ijerph18042107

**Published:** 2021-02-22

**Authors:** Justyna Grudziąż-Sękowska, Monika Zamarlik, Kuba Sękowski

**Affiliations:** 1 Centre of Postgraduate Medical Education, School of Public Health, 01-813 Warsaw, Poland; 2 Faculty of Health Sciences, Institute of Public Health, Jagiellonian University, 31-007 Kraków, Poland; monika.zamarlik@doctoral.uj.edu.pl; 3 Doctoral School, Law College, Kozminski University, 03-301 Warsaw, Poland; kuba.sekowski@gmail.com

**Keywords:** children, diabetes mellitus, type 1, health status disparities, social inequalities

## Abstract

Type 1 diabetes mellitus (T1D) is, next to obesity and asthma, the most common chronic disease in children in Poland. T1D is not only a medical challenge, but it also affects all areas of a sick child’s life and family functionality. New forms of therapy facilitate the daily management of the disease, but their availability is limited and partly dependent on socioeconomic status. This study aimed to assess the incidence and interrelationships between the child’s health condition and the applied therapy model, and selected aspects of the child’s family functionality and access to health and care services. The survey involved 206 child and youth caregivers with T1D who are members of Facebook support groups. The analysis of the obtained results revealed the existence of links between family income level and the type of insulin therapy applied. Children from families with a better financial situation (subjective and objective) were more likely to have additional medical consultations and make more frequent control visits. In families with a higher level of income, the T1D-induced restriction of child activity was less frequent. Living outside of urban centers was associated with a reduced availability of care or educational facilities adapted to take care of a child with T1D. No statistically significant correlations were observed between demographic and economic factors and the child’s health status expressed by the occurrence of complications. The incidence of the latter, however, affected the child’s family situation.

## 1. Introduction

Type 1 diabetes (T1D) is an immune-mediated disease. It is a disorder characterized by the progressive destruction of beta cells in the pancreas, leading to the cessation of insulin secretion and subsequent hyperglycemia [1,2]. The exact cause of the destruction of pancreatic β cells is not fully known, but some factors are indicated that initiate their destruction by the immune system. These include not only genetic factors [3,4], but also environmental factors (including rubella viruses, mumps viruses, cytomegaloviruses, and the consumption of cow’s milk protein) [5]. T1D is a chronic disease that requires a constant external insulin supply.

Despite technological advances in pharmacotherapy, insulin delivery devices, and glycemia measurement, T1D treatment presents patients and their families with challenges that affect most aspects of their daily lives [6]. Maintaining adequate metabolic control to minimize the risk of diabetes-related complications while maintaining a flexible lifestyle and quality of life is a particular challenge for the youngest patients—children and adolescents.

The last few decades have seen a significant increase in the incidence of T1D worldwide. This corresponds to an increase of over 120,000 new T1D cases in children and adolescents up to 19 years of age per year. The largest increase is in the population of the youngest children (<5 years), for whom living with the disease will be the greatest burden. In Europe, there has been an overall annual increase in the incidence of T1D of 3.9% recorded, and the incidence rate has been projected to double for children <5 years old between 2005 and 2020. [7]

The increasing prevalence of T1D in developed countries is worrying because T1D negatively affects quality and life expectancy, mainly due to the diseases and deaths resulting from its chronic complications [8,9]. Children from low-income families with an unfavorable socioeconomic status are more likely to suffer from the adverse course of the disease, worse glycemic control, and less access to new, expensive medical technologies, and, thus, incur a higher risk of health complications [10].

In 2018 in Poland, nearly 22,000 people under 18 years of age were suffering from T1D. This constituted 3.17% of the underage population (a 2.5% increase compared to 2013) [11]. In some regions of Poland, the T1D maturity rate in the 0–4 age group increased by more than 2.5 times. [12]. In Poland, most of the latest treatment technologies are available to facilitate the daily functioning of T1D patients. However, not all of them are reimbursed under the general health insurance system. In Poland, there is only one public payer (the National Health Fund). A significant proportion of medicinal products (including insulin and accessories for personal insulin pumps, as well as continuous glycemic monitoring (CGM) systems) require partial payment by the patient. There is no private (supplementary or complementary) health insurance covering pediatric diabetology

In 2018, 18,900 thousand patients benefited from treatment with a personal insulin pump (a 60% increase compared to 2013) [11]. Since 2018, CGM technologies have been partially refunded for persons up to 26 years old. During the first year, 3100 patients gained access to CGM technology. The level of reimbursement by the public payer was 68.9% on average (16 million PLN, approximately 4 million EUR) [11]. The remaining costs of the CGM systems were borne directly by patients (via out of pocket payments).

Considering its level of economic development, Poland is not a country with gross economic inequalities. Their level of economic inequality is lower than that in other Central and Eastern European countries undergoing a similar process of economic transformation. At the same time, income disparities in Poland are high compared to richer EU countries [13]. In Poland, there are also significant deficiencies in terms of social infrastructure. The insufficient availability of institutional forms of childcare services (nurseries and kindergartens) and their inadequacy to care of children with chronic diseases (including T1D [14]) limit the ability of children and adolescents with T1D to lead a life appropriate for their healthy peers and constitute a significant burden for their families [15]. The need for personal care of a sick child is a significant burden for caregivers, often leading to a reduction in their professional activity and threatening to lower their socioeconomic status.

Therefore, it becomes important to assess the overall quality of life of children with T1D, taking into account the impact of the disease on their ability to function in society and their family situation, including their socioeconomic status [16,17,18,19].

Previous studies often used the diabetes-specific Diabetes Quality of Life for Youth (DQOLY) questionnaire [20]. That questionnaire provides information on the quality of life specific to teenagers with diabetes [21]. To assess the quality of life of a wider population of T1D children, the Pediatric Quality of Life Inventory (PedsQL) 3.0 Diabetes Module was developed [22,23,24]. It allows for the comparison of the quality of life of children with T1D with that of a population of healthy children. These tools have enabled a reliable assessment of the quality of life of children with T1D [22]. The fundamental part of the evaluation remains valid with regard to Poland [25]. The results obtained with the use of these quality of life assessment tools are often correlated with data on applied therapy and its outcomes, e.g., metabolic control, and number of hypo- and hyperglycemic episodes [26,27]. However, there are no studies on the access of children and young people with T1D to health benefits and care services, which is a problem in Poland [15].

The aim of the study was to assess the incidence and interrelationships between the child’s health condition and the applied therapy model, and selected aspects of the child’s family functionality and access to health and care services. For this purpose, a specially designed questionnaire was used which, in addition to the information used in DQOLY and PedsQL about the most recent hemoglobin A1c (HbA1c) levels, included questions about the impact of the disease on selected aspects of the child’s family functionality and access to health benefits and care services.

Due to the important, constantly growing role of the Internet—as a place of searching for information, contact, and support for patients with T1D [28,29]—the survey was conducted among social media users associated in groups with families of children and adolescents with T1D.

The research program under which the study was conducted received a positive assessment from the IRB of the Centre of Postgraduate Medical Education (no. 501-4-44-28-18). All the procedures performed in the study involving human participants were in accordance with the ethical standards of the institutional and national research committee, as well as with the 1964 Helsinki Declaration and its later amendments or comparable ethical standards.

## 2. Materials and Methods

### 2.1. Data Collection

The research material consisted of the data collected through questionnaires addressed to the caregivers of children diagnosed with type 1 Diabetes. The survey was conducted in the autumn of 2019 through Facebook groups of T1D caregivers: “Let’s help each other” type 1 diabetes mellitus and “Let’s get the colors of life back” type 1 diabetes mellitus. These groups have a total of more than 17,000 members who have one or more T1D children in their care (which functioned as an inclusion criterion). The group members were invited to fill in the questionnaire in electronic form (CAWI). Before completing the questionnaire, the participants were informed about the scope and purpose of the study, as well as about the voluntary and anonymous nature of the answers provided. The study (questionnaire) was not connected with any intervention (diagnostic or therapeutic), nor did it constitute a part of such an intervention. Therefore, according to Polish regulations on the provision of health services, obtaining qualified informed consent was not required.

To avoid multiple completions of the survey by the same respondents who could belong to both of the indicated groups, duplicate records were removed from the obtained answers. The criteria for qualifying the record as recurring were the same answers to questions about the child’s name and date of birth. Records for people over 18 years have also been deleted (as an exclusion criteria). Any information on persons subject to exclusion was not subsequently processed and was removed from the dataset. In accordance with the applicable regulations on personal data protection, the survey did not include information that could identify the respondent (caregiver) or the child they cared for.

**Figure 1:**
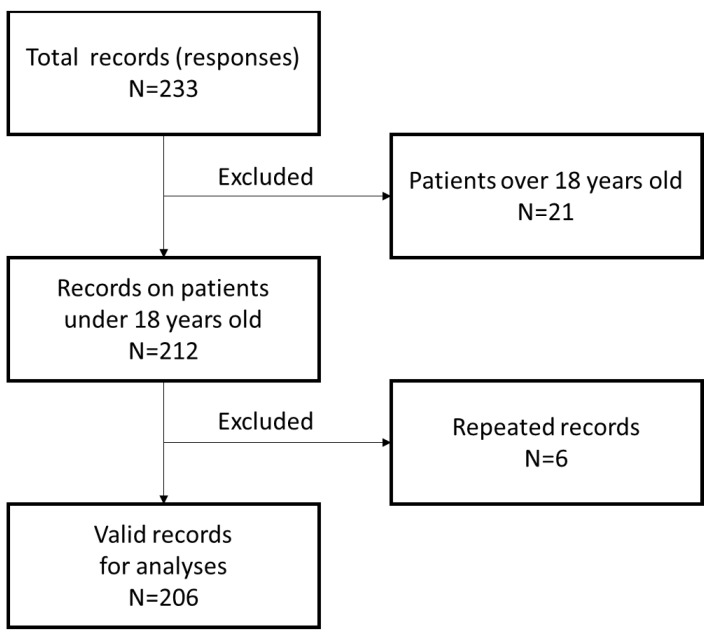
Flow chart of data collection.

In total, 233 complete questionnaires were completed. After eliminating the questionnaires concerning patients over 18 years of age, 212 questionnaires were obtained. After eliminating the duplicates, 206 questionnaires were received (Figure 1).

### 2.2. Variables

The survey questions were divided into four modules (groups of variables), concerning, respectively:

1. Demographic data of the child and parent/caregiver—module I;
2. Information on the child’s health and therapy—module II;
3. Information about the impact of the disease on family and child life—module III;
4. Information on the provision of care for the child by other persons or institutions—module IV.

#### 2.2.1. Demographic Data—Module I

The demographic dataset included: the child’s age (in full years); the child’s gender; the child’s family situation (full family—a family with two adults raising a child/children; single-parent family—a family with one adult raising a child/children; other—in the case of raising a child outside the biological or adoptive family); primary caregiver—the person caring for the child in the context of the disease (mother or father—also, in the case of adopted children, relative, another person—caregiver); primary caregiver age (in full years); the child’s place of residence (a village, a town with up to 20,000 inhabitants, a town with 20,000–100,000 inhabitants, a city with 100,000–500,000 inhabitants, a city with over 500,000 inhabitants); subjective assessment of the economic situation of the family (using a five-point Likert scale); and objective assessment of the family’s economic situation (income per person in the household at intervals of PLN 500/EUR 110).

#### 2.2.2. Health Status—Module II

Data on the child’s health status included: the age of onset of T1D (in full years); the model of insulin therapy used (CSII or MDI); the type of doctor treating the child (diabetologist, GP, or other doctor); the frequency of visits to the attending physician (one, two, three, or four or more times a year); regularity of visits to the attending physician (regularly, irregularly at the will of the child’s caregiver or irregularly due to the limited availability of visits); specialist consultations in the last 12 months (with an ophthalmologist, neurologist or other doctor); specialist consultations during the last 5 years or since the disease (with an ophthalmologist, neurologist or other physician); and T1D complications diagnosed in the child (none, ophthalmologist, nephrologist, or dermatologist).

#### 2.2.3. Impact on Child’s and Family’s Life—Module III

The impact of the child’s disease on the situation of this child and their family was assessed in relation to the following areas: changes in the professional activity of the caregivers and its reasons; changes in the family situation (it was possible to indicate more than one answer describing changes in the division of responsibilities, financial situation, and emotional relations in the family), concerns about the disease in the caregiver and/or child (it was possible to indicate more than one statement describing the feelings of the caregiver and the child) and the amount of expenses related to the child’s therapy (the level of monthly private expenses at intervals of PLN 100/22EUR).

#### 2.2.4. Impact on Child’s and Family’s Life—Module III

A child’s illness can cause social exclusion, which first affects the child and then affects their family. The following variables were used to assess the occurrence of symptoms of social exclusion associated with developing T1D: the possibility of entrusting childcare to another person during the day, the possibility of entrusting such care during the night, the child’s attendance at care/educational institutions (nursery, kindergarten, school), and the reasons for not attending such institutions.

### 2.3. Statistical Analysis

The collected data were subjected to statistical analysis. In order to determine the characteristics of the surveyed population, descriptive statistics tools were used, and the Pearson Chi-square test and Cramer’s V coefficient were used to assess the relationship between individual variables. Statistical analysis was performed in the IBM SPSS Statistics version 26 program (IBM Corp., Armonk, NY, USA) with the statistical significance level *p* < 0.05.

## 3. Results

### 3.1. Participants Characteristics

Information was collected on 206 T1D children (Table 1), 109 (52.9%) of whom were girls and 97 (47.1%) were boys. These groups did not differ in terms of the presence of the examined factors. The average age of the children was 7.91 years (SD = 4.45). Most of the children (>90%) lived in cities, more than half of whom lived in big cities and the largest cities. 66.5% of the respondents were brought up in complete families, and less than 2% (three cases) were brought up outside of their families.

Due to the size of the sample (N = 206), the groups of respondents distinguished on the basis of some of the demographic characteristics (particularly place of residence and family situation combined with gender and age) were so small as to make it impossible to obtain statistically significant results showing their association with data from the other modules of the questionnaire. Statistically significant results were obtained only when the whole sample was analyzed. This was the case, for example, for the revealed associations between family type and the level of concern of the child and the caregiver.

### 3.2. Correlations between Groups of Variables (Modules)

A number of statistically significant correlations between pairs of variables from individual modules were identified. The strength of association of these variables—as measured by Cramer’s V coefficient—was varied, and ranged from 0.808 (signaling a very strong relationship) to 0.140 (signaling a weak relationship). The greatest number of correlations was observed between the variables from module I (demographic data) and the variables from module III (the impact of the disease on the life of the child and family); 19 statistically significant correlations out of 49 (38%) possible correlations between pairs of variables were found with these modules. There was an association between the variables from module III and the variables from module II (data on health and treatment); 15 statistically significant correlations out of 56 (26.8%) possible associations were present. The variables from module IV (data on the possibility of providing care outside the child’s family) were related to the variables from module I in 5 out of 21 (23.8%) possible cases, and with the variables from module II in 4 out of 24 (16.7%) possible cases. Numerous links were also observed between the variables characterizing the child’s demographic situation (module I) and those describing their health and treatment (module II).

There were statistically significant relationships between 16 out of the 56 (28.6%) pairs of variables from these modules. Correlations also occurred between the variables belonging to individual modules. These correlations were, respectively: 6 out of 21 (28.6%) cases in module I, 26 out of 28 (92.8%) cases in module II, 16 out of 21 (76.2%) cases in module III, and 2 out of 3 (66.6%) cases in module IV.

#### 3.2.1. Correlation between Demographic Factors and Health Status

The average income per person in the family was the factor with the highest number and strength of correlation with the data illustrating the health condition and treatment of the child. The income level correlated most strongly with the type of insulin therapy applied. The average monthly income per family member of all families of children using MID (58 cases) was in the four lowest ranges (<PLN 2,000, approx. EUR 500). Among those using insulin pumps, this level of income was found in 48% of cases. Children from families with a better financial situation (subjective and objective) used additional medical consultations more often and made more frequent control visits. The child’s place of residence influenced both the choice of insulin therapy method and the use of additional specialist consultations. No statistically significant correlation was observed between the child’s demographic situation and the occurrence of complications or the main physician.

Detailed information on the number and strength of the observed connections is presented in Table 2.

#### 3.2.2. Influence of Demographic Factors

The analysis of the collected data indicates a correlation between the occurrence of specific demographic factors and the values of the variables characterizing the impact of the disease on the life of the child and their family, and the possibility of taking care of the child outside the family. The subjective assessment of the family’s financial situation was related to the level of expenses incurred from the child’s therapy and the occurrence of anxieties related to the child’s disease, which was also negatively correlated with the limitation of the child’s activity caused by T1D. Similar correlations were observed in the case of the variable describing the average income per person in the family (an objective measure of the family’s economic situation), which was also associated with the frequency of follow-up visits to the attending physician. Similar relationships occurred with regard to the child’s place of residence, which also affected the frequency of attendance at care or educational institutions.

The strength of the relationship between demographic factors and other variables varied. Cramer’s V-value ranged from 0.157 in the case of the association of the child’s family type with the regularity of control visits to 0.808 in the case of the child’s age and the possibility of entrusting daytime care to a third party (other than the permanent caregiver).

Detailed information on the number and strength of the observed connections is presented in Table 3.

### 3.3. Influence of Health Status

Significantly fewer connections were observed between the current health condition and the family situation of the child. The amount of health care expenses (out of pocket payments) was related to the type of insulin therapy applied (with the V-value of the Cramer’s coefficient being 0.558) and the frequency of follow-up visits to attending physicians of other specialties in the last 12 months and 5 years (with the V-value of the Cramer’s co-ordinates as 0.588 and 0.592, respectively). The child’s age was important in the case of the possibility of entrusting childcare to persons other than parents (caregivers). The lack of such a possibility concerned mainly the youngest children—those under the age of compulsory schooling (6 years). The average age of children whose parents could not count on this form of support from other people or institutions (n = 24) was 2.79 years.

Detailed information on the number and strength of the observed connections is presented in Table 4.

## 4. Discussion

The aim of the study was to determine the degree of prevalence of selected problems in the functionality of the family of a child with T1D. The study covered the difficulties in accessing health and care services for children and young people with T1D in Poland. The relationship of these problems with socioeconomic status (SES) and the health condition of the respondents was also assessed.

The number of caregivers of children and adolescents with T1D who took part in the study (206 people) was significant, as it amounted to almost 1% of the total number of people under 18 years of age suffering from T1D in Poland (about 22,000). For this reason, the obtained results may reflect the situation of this social group as faithfully as possible.

The studies conducted so far show that low SES is associated with a number of risk factors among T1D patients, such as: poor glycemic control [30,31,32], smoking [33,34], hypertension [34,35], and an increased number of hospitalizations [36]. They also contribute to an increased risk of death [37,38,39,40] in the T1D population.

A diagnosis of T1D at a young age, and therefore a long disease period, may intensify the effect of these factors. Among children and adolescents with T1D, it was observed that low SES was accompanied by worse glycemic control results and compliance with therapeutic adherence [41,42]. The results of the conducted research confirm these observations. The groups of respondents with higher and lower income (both subjectively assessed and in absolute terms) clearly differed in the use of modern forms of therapy (Personal insulin pumps), as well as medical check-up visits, aimed at early diagnosis of possible T1D complications (ophthalmological, nephrological and neurological consultations). The spatial distribution of poverty in Poland may partially explain this relationship. This problem occurs most strongly in small and medium-sized towns, and to a lesser extent in villages, large cities and the largest cities [43]. The results of the study indicate that the place of residence was associated with a similar number of variables describing the patient’s health, family situation, and access to care and educational services (seven associations), as was income level (six associations). However, these relationships were often of a weaker nature (with a lower Cramer’s V coefficient).

The importance of the income level, when assessed objectively in particular, is shown by its connection with the applied form of insulin therapy and the use of specialist consultations. This may be due to the fact that PIP therapy, similarly to the use of CGM, is only partially reimbursed under general health insurance. The use of PIP and CGM therefore imposes higher caregiver fees. This assumption is confirmed by the observed strong correlation of these phenomena with the declared level of private expenditure on health care (out of pocket payments). Similar results were obtained in studies carried out in Germany and Canada concerning, respectively, the choice of treatment with insulin pumps [42] and the willingness to pay for better T1D therapy [44,45].

The vast majority of therapeutic recommendations assume that a patient with T1D should consult with a doctor at least every 3–4 months [46]. A similar recommendation is formulated by the Polish Diabetes Society [47]. It is also advisable for the patient to undergo specialist consultations (ophthalmological, nephrological, and neurological). Adherence to these recommendations is essential for the success of the treatment (primarily expressed in better glycemic control). Following these recommendations also has the potential to reduce the SES-induced gap [48,49]. Therefore, the low average number of visits to the attending physician (2.7 visits per year) and specialist consultations (less than 0.2 in the last 12 months and about 0.25 in the last 5 years) is of concern. Taking into account the recommended frequency of such consultations (ophthalmological consultations in particular), as well as the average age of patients (7.9 years) and time from the diagnosis of the disease (approximately 3 years) in the study population, we should expect these values to be several times higher. A partial explanation of the observed situation may be the general problems with the availability of certain benefits in the public health care system in Poland [50,51]. In this context, the low percentage of children and adolescents, as well as their caregivers, who express concerns about their health and the possibility of complications, is surprising. This may be due to insufficient knowledge of underage patients and their caregivers about the disease itself and of the importance of complying with therapeutic recommendations [52,53].

The study did not confirm the existence of a relationship between the place of residence and the availability of care services (e.g., a nursery) and education services (kindergarten) for children under compulsory school age (up to 6 years of age). On the other hand, the results of this study indicate that the connection of the parents’ possibility of entrusting another person (guardian) or institution (nursery, kindergarten) with caring for a child suffering from T1D is related to the child’s age. This situation may be caused by the lack of preparation of people and institutions to meet the requirements of caring for a child with T1D [54], as well as by the lack of appropriate legal regulations that would require the admission of such a child or ensure that their special needs are taken into account during their stay in the facility.

**Figure 2:**
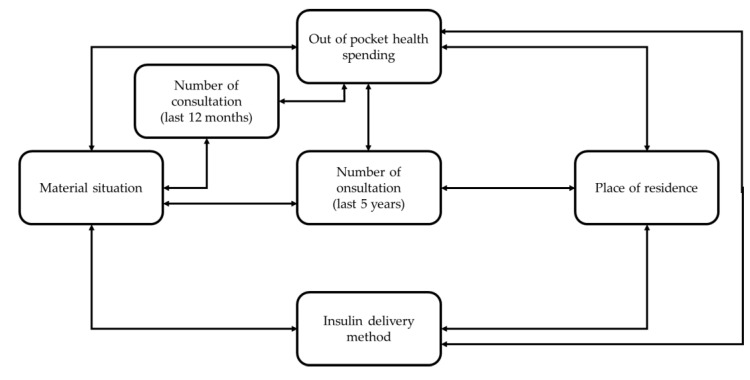
Strongest correlations.

The strongest correlations between observed variables are presented in Figure 2.

## 5. Study Limitations

This study was a preliminary research (exploratory study). It was conducted on a smaller-than-optimal sample of respondents, whose selection (membership in one of two Facebook self-help groups) was not random. The size of the sample made it impossible to conduct a more in-depth statistical analysis, in particular one that could illustrate potential further differentiation of the situation of individual groups of children and adolescents with T1D in Poland.

## 6. Conclusions

Subject to the limitations outlined in the previous section, the conducted research shows the existence of strong links between income level, the availability of modern therapy methods (insulin pumps, CGM systems), and the possibility of using specialist consultations for children and adolescents with T1D in Poland. The nature of the study does not allow a determination as to whether this relationship is direct or whether income level interacts with other factors, such as the level of education or place of residence.

The discrepancy of the declared model of diabetes care with the current scientific knowledge and recommendations of medical societies raises concerns.

Unequal access to modern forms of therapy and the suboptimal nature of the actually implemented care regimen may have negative effects on the health of the population of children and adolescents with T1D. Therefore, the factors influencing this state of affairs should be the subject of further research

It is advisable to carry out a further, in-depth analysis of the situation of children with T1D in Poland. The results of such a study (optimally with a much larger random sample) could be an important contribution to the formation of public policies in the field of health care and social policy, as well as an aid in everyday clinical practice.

## Figures and Tables

**Table 1:** Demographic characteristics of participants (N = 206).

Characteristics	Villages and Towns N = 19	Small Cities (<20,000 Inhabitants) N = 32	Medium Cities (20,000–100,000 Inhabitants) N = 46	Big Cities (100,001–500,000 Inhabitants) N = 81	Largest Cities (>500,000 Inhabitants) N = 28
Child’s age (years)	7.74 (4.68)	9.22 (4.46)	8.00 (4.37)	7.33 (4.48)	8.07 (3.96)
Caregiver’s age (years)	41.63 (8.30)	43.31 (9.69)	41.65 (10.08)	40.07 (9.90)	38.71 (8.44)
Child’s family situation					
Two-parent family (cases)	14	24	33	50	16
One-parent family (cases)	5	8	13	29	11
Other (cases)	-	-	-	2	1

**Table 2:** Correlation between demographic factors and health status.

Cramer’s V (*p*-Value)1	Child’s Age	Caregiver’s Age	Family Type	Main Caregiver	Place of Residence	Material Situation (Self-Assessed)	Material Situation (Objectively)
Child’s Health and Therapy—Module II							
Frequency of control visits	-	-	-	0.343 (<0.001)	-	-	0.251 (0.027)
Age at diagnosis	0.687 (<0.001)	0.354 (<0.001)	-	0.354 (<0.001)	-	-	-
Insulin delivery method	-	-	-	-	0.564 (<0.001)	0.588 (<0.001)	0.771 (<0.001)
Regularity of control visits	-	-	0.157 (0.038)	-	-	-	-
Attending physician	-	-	-	-	-	-	-
Consultation (last 12 months)	-	-	-	-	0.507 (<0.001)	0.561 (<0.001)	0.653 (<0.001)
Consultation (last 5 years)	-	-	-	0.274 (0.004)	0.529 (<0.001)	0.586 (<0.001)	0.643 (<0.001)
Occurrence of complications	-	-	-	-	-	-	-

Correlations marked with (-) were statistically insignificant (*p* > 0.05).

**Table 3:** Influence of demographic factors.

Cramer’s V (*p*-Value)1	Child’s Age	Caregiver’s Age	Family Type	Main Caregiver	Place of Residence	Material Situation (Self-Assessed)	Material Situation (Objectively)
Child’s and Family’s Life—Module III							
Changes in professional activity	-	-	-	-	-	-	-
Limited access to activities	-	-	-	-	0.224 (0.003)	0.242 (<0.001)	0.304 (<0.001)
Change of family situation	-	-	-	0.321 (0.018)	-	-	-
Out of pocket health spending	-	-	0.185 (0.029)	-	0.682 (<0.001)	0.737 (<0.001)	0.493 (<0.001)
Caregiver’s concerns	0.321 (0.007)	0.240 (0.040)	0.219 (0.006)	-	0.239 (0.042)	0.254 (0.011)	0.252 (0.001)
Child’s concerns	0.414 (<0.001)	0.366 (<0.001)	0.184 (0.030)	-	-	-	-
Support Sources	0.332 (0.006)	-	-	0.321 (0.018)	-	-	-
**Provision of Care—Module IV**							
Day-time care (3rd person)	0.808 (<0.001)	0.391 (<0.001)	-	-	-	-	-
Night-time care	-	-	-	-	-	-	-
Access to institutional care	0.762 (<0.001)	0.433 (<0.001)	-	-	0.314 (0.037)	-	-

Correlations marked with (-) were statistically insignificant (*p* > 0.05).

**Table 4:** Influence of health status.

Cramer’s V (*p*-Value)1	Age at Diagnosis	Insulin Delivery Method	Main Doctor	Frequency of Control Visits	Regularity of Control Visits	Consultations (Last 12 Months)	Consultations (Last 5 Years)	Occurrence of Complications	
Child’s and Family’s Life—Module III									
Changes in professional activity	-	0.141 (0.043)	-	0.255 (0.004)	-	-	-	-	
Limited access to activities	-	-	-	-	-	0.230 (0.001)	0.217 (0.003)	-	
Change of family Situation	-	-	-	-	-	-	-	0.430 (0.001)	
Out of pocket health spending	-	0.558 (<0.001)	-	0.244 (0.044)	0.261 (0.032)	0.588 (<0.001)	0.592 (<0.001)	-	
Caregiver’s concerns	0.235 (0.003)	-	-	-	-	0.140 0.044)	-	-	
Child’s concerns	0.408 (<0.001)	-	-	-	-	-	-	-	
Support sources			0.349 (<0.001)	0.258 (0.039)	-	-	-	-	
**Provision of Care—Module IV**									
Day-time care (3rd person)	0.592 (<0.001)	-	-	-	-	-	-	-	
Night-time care	0.241 (0.046)	-	0.259 (0.001)	-	-	-	-	-	
Access to institutional care	0.628 (<0.001)	-	-	-	-	-	-	-	

Correlations marked with (-) were statistically insignificant (*p* > 0.05).

## Data Availability

The data presented in this study are available on request from the corresponding author. The data are not publicly available due to technical reasons.
